# Atomic-resolved depth profile of strain and cation intermixing around LaAlO_3_/SrTiO_3_ interfaces

**DOI:** 10.1038/srep28118

**Published:** 2016-06-15

**Authors:** H. Zaid, M. H. Berger, D. Jalabert, M. Walls, R. Akrobetu, I. Fongkaew, W. R. L. Lambrecht, N. J. Goble, X. P. A. Gao, P. Berger, A. Sehirlioglu

**Affiliations:** 1 MINES ParisTech, PSL Research University, MAT - Centre des matériaux, CNRS UMR 7633, BP 87 91003 Evry, France; 2 Université Grenoble Alpes, INAC-SP2M, LEMMA, F-38000 Grenoble, France; 3 CEA, INAC-SP2M, LEMMA, F-38000 Grenoble, France; 4 Laboratoire de Physique des Solides, Université Paris Sud, Bât 510, 91405 Orsay, France; 5 Departments of Materials Science and Engineering, Case Western Reserve University, Cleveland, Ohio, USA; 6 Departments of Physics, Case Western Reserve University, Cleveland, Ohio, USA; 7 NIMBE, CEA, CNRS, Université Paris-Saclay, CEA Saclay 91191 Gif sur Yvette, Cedex, France

## Abstract

Novel behavior has been observed at the interface of LaAlO_3_/SrTiO_3_ heterostructures such as two dimensional metallic conductivity, magnetic scattering and superconductivity. However, both the origins and quantification of such behavior have been complicated due to an interplay of mechanical, chemical and electronic factors. Here chemical and strain profiles near the interface of LaAlO_3_/SrTiO_3_ heterostructures are correlated. Conductive and insulating samples have been processed, with thicknesses respectively above and below the commonly admitted conductivity threshold. The intermixing and structural distortions within the crystal lattice have been quantitatively measured near the interface with a depth resolution of unit cell size. A strong link between intermixing and structural distortions at such interfaces is highlighted: intermixing was more pronounced in the hetero-couple with conductive interface, whereas in-plane compressive strains extended deeper within the substrate of the hetero-couple with the insulating interface. This allows a better understanding of the interface local mechanisms leading to the conductivity.

The pioneering work by Ohtomo and Hwang^1^ reported the formation of an electron gas with a large charge carrier density at the interface between two band insulators, LaAlO_3_ (LAO) film on SrTiO_3_ (STO) substrate. However the mechanisms of charge transfer and transport in this system are still not clearly established. This first observation of a metallic sheet formation was attributed to a decrease in valence of Ti cations located in the first unit cell of STO. This led Ohtomo and Hwang to propose an initial hypothesis involving the build-up of an electrostatic field across the thickness of the polar LAO film deposited on the non-polar STO substrate. It was suggested that the electrostatic field was screened by lattice polarization up to 3 unit cell (u.c.) size films. An electronic reconstruction was assumed to occur when a thickness of four unit cells was reached, allowed by a charge transfer from the LAO film surface to Ti cations just below the interface. Since then, some discrepancies between the charge carrier densities and mobilities predicted by this model and those measured experimentally have been reported^2^,^3^,^4^, as has the high sensitivity of the conductivity to growth parameters^2^,^3^,^4^,^5^,^6^,^7^,^8^,^9^,^10^,^11^,^12^,^13^,^14^,^15^. This broadens the field of possible mechanisms responsible for the development of this surprising metallic behavior. Other structural and chemical changes have been revealed near the interface, including the dilatation of STO cells and polar distortions^16^,^17^,^18^,^19^,^20^,^21^,^22^,^23^, cation intermixing^14^,^22^,^24^,^25^,^26^,^27^, oxygen vacancies^12^,^24^,^28^,^29^,^30^ and lanthanum deficiency in the film^31^. The specific roles of these local modifications on the interfacial conduction are yet not fully understood.

The objective of this paper is to provide insight into the role of intermixing and local structural distortions on charge transport. We approach this by correlating the chemical and strain profiles of two heterostructures prepared in the same experimental conditions above and below the critical thickness of four unit cells for the insulator/metal transition. The presumed confinement of the charge carriers in a sheet with a thickness of a few unit cells requires an analytical tool with a depth resolution below the cell dimension (~0.4 nm). Medium-Energy Ion Scattering (MEIS) offers this depth resolution due to the high energy loss of medium-energy ions when interacting with solids. This ion spectroscopy has been recently used in LAO/STO systems to demonstrate qualitatively cation intermixing^14^,^26^. MEIS was also used to establish strain profiles in non-oxide semiconductor nano-objects^32^,^33^. Here we present, for the first time, quantitative profiles of the strontium and lanthanum concentrations correlated with profiles of the cell parameter variations around these atoms for both insulating and conductive hetero-structures. Epitaxial strains taking into account the chemical gradients cannot explain the magnitude of cell distortions measured. Charge defects have to be considered, with distinct spatial distributions between the insulating and conducting heterostructures.

## Results and Discussion

### Interface characterizations

#### Interface morphology, Ti valence and oxygen vacancies

**Figure 1:**
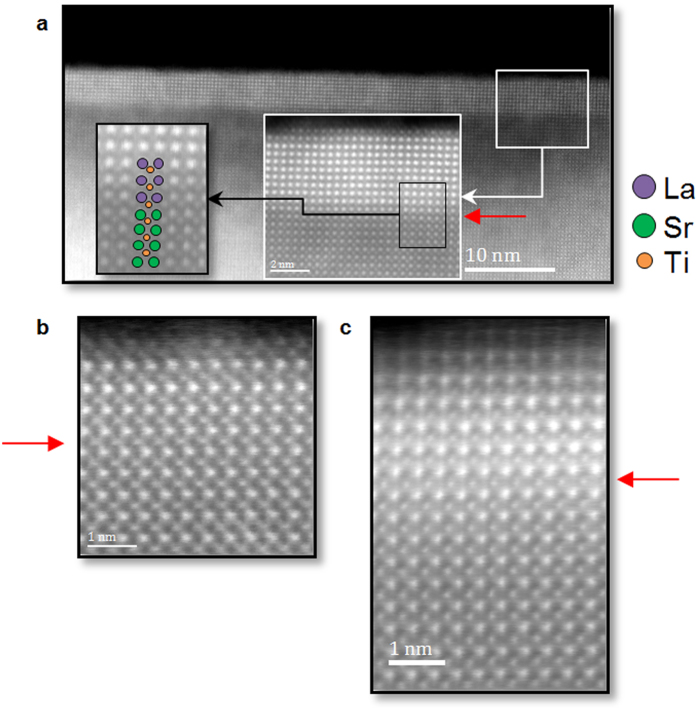
Representative HAADF images of LaAlO_3_ films grown pseudomorphically on SrTiO_3_. HAADF of (**a**) 10 u.c. (**b**) 3 u.c. (**c**) 5 u.c.-thick films deposited on a SrTiO_3_ substrate. The same process conditions were used to deposit the films. The interfaces are shown by a red arrow. No misfit dislocations could be detected at LAO/STO interfaces along the entire distance observed via Nion UltraSTEM. The left inset in (**a**) highlights the off-center displacement of Ti near the interface.

Epitaxial films with 3 and 5 u.c. thickness were grown by Pulsed Laser Deposition. The oxygen partial pressure during deposition was fixed at 10^−4^ Torr, and the temperature reached 750 °C. The parameter misfit between the substrate (a_STO_ = 3.905 Å) and the film (a_LAO_ = 3.791 Å using a pseudo cubic description) did not induce strain relaxations via interfacial misfit dislocations as no dislocations could be detected along the foil observed for both samples, as seen on Fig. 1.

The 5 u.c. sample exhibited a conductive interface while for the 3 u.c. film the resistance exceeded our instrumental limits (>100 MΩ) (Electrical measurements are reported in the Supplementary Fig. S1). Thus, consistently with previous studies^4^,^14^,^34^,^35^,^36^, the critical thickness lies between 3 and 5 u.c. These samples are good candidates to investigate structural and/or chemical differences between conductive and insulating samples.

**Figure 2:**
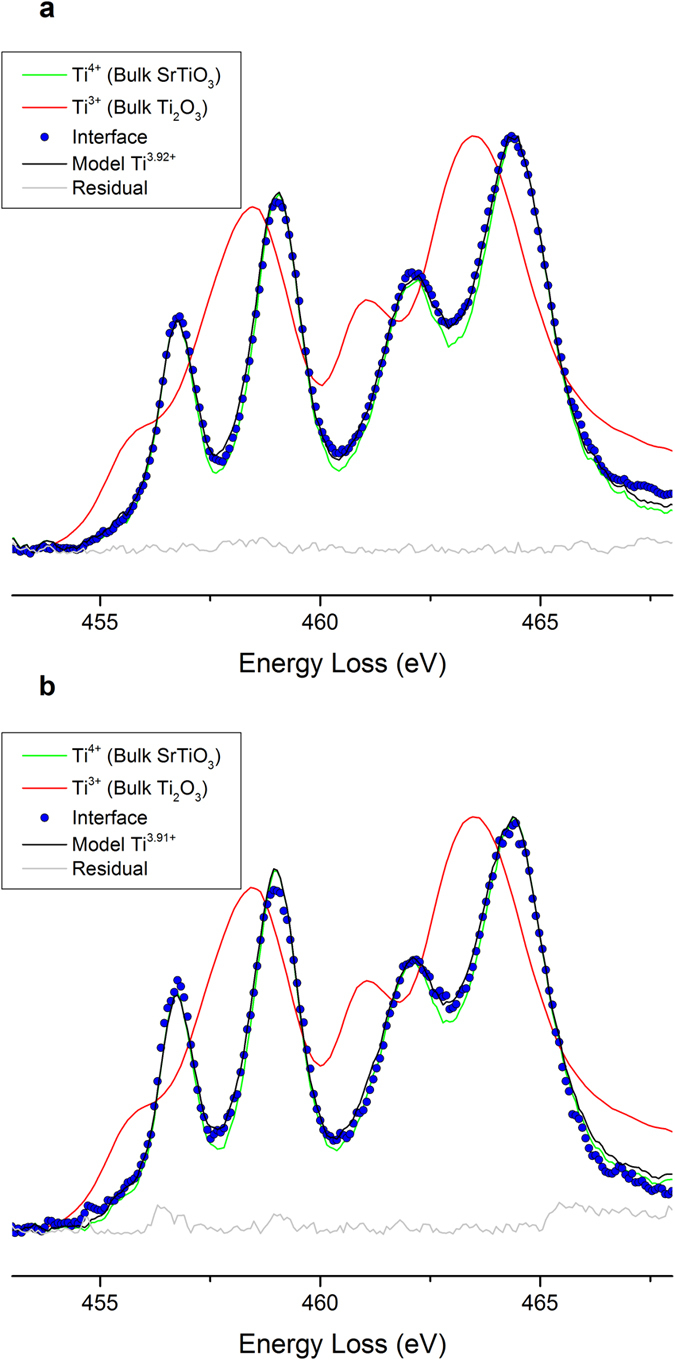
Ti-L_2,3_ edge EELS taken at the LaAlO_3_/SrTiO_3_ interface. EELS spectrum at the interface of the (**a**) 3 u.c. (**b**) 5 u.c. sample. The contributions of Ti^4+^ and T^3+^ to the Ti edge were deduced from a linear combination of two reference spectra, a green one for Ti^4+^ (SrTiO_3_ away from the interface of the (**a**) 3 u.c. (**b**) 5 u.c. sample) and a red one Ti^3+^ (Bulk Ti_2_O_3_) recorded on the same spectrometer. The method of least squares has been used to fit the experimental and the simulation spectra. The blue dots spectra correspond to the experimental EELS measurement at the very first unit cell below the interface. The black curves represent the best simulation that fitted the experimental spectrum. One observes that the Ti oxidation state is quite similar between the conductive and insulating samples grown in the same conditions.

In the original hypothesis from Nakagawa *et al*.^24^ the diverging electrostatic potential in the growing polar film is removed by the transfer of half an electron per unit cell into the first STO layer below the interface. A theoretical average valence of Ti^3.5+^ would be thus expected resulting in a 2D carrier density of 3.3 × 10^14 ^cm^−2^. Electron Energy Loss Spectroscopy (EELS) profiles across the interface of the selected samples were used to deduce the contribution of Ti^3+^ to the Ti-L_2_L_3_ absorption edges. A minimum valence of Ti^3.9+(+/−0.05)^ was found located in the first unit cell below the interface of both samples (Fig. 2). This would lead to a maximum theoretical density of free charge carriers of 6.6 × 10^13^ (+/−3.28 × 10^13^) cm^−2^ if we assume that all the carriers originate from Ti^4+^ reduction. Experimental measurements of Hall coefficient on the 5 u.c. sample below 10 K (reported in the Supplementary Information) revealed a 2D charge carrier density (≈3 × 10^14 ^cm^−2^) that was comparable to theoretical density (3.3 × 10^14 ^cm^−2^). However, 2D charge carrier density at room temperature (n > 1.2 × 10^15 ^cm^−2^), was much higher than the density calculated based on EELS valence measurements. This suggests that the conduction was not purely bidimensional. The hypothesis of a quasi 2D conduction zone restricted to the first layers above and below the interface, still underestimates the charge carrier density with respect to the Hall measurements (EELS measurements of Ti oxidation state measured 1 u.c. above and under the interface are presented in the Supplementary Fig. S2). This would confirm the three dimensionality of the conduction. This delocalization of the carriers normal to the interface was suggested to explain the weak contribution of Ti^3+^ on the EELS measurements at the interface^29^,^37^,^38^. The similar Ti valences measured below the interfaces in the conducting and insulating samples does not play in favor of charge injection by a polar catastrophe^29^,^38^,^39^, at least for such film thicknesses. This highlights the role of the carrier mobility to explain the observed discrepancies in terms of electrical conduction.

**Figure 3:**
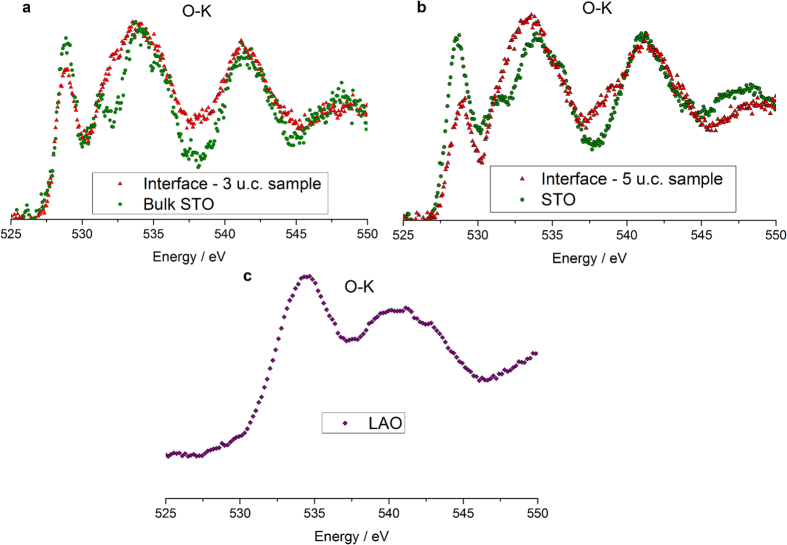
EELS fine structure of the O-K absorption edge across two LaAlO_3_/SrTiO_3_ heterostructures. (**a**,**b**) Experimental spectra recorded at the (**a**) 3 u.c. (**b**) 5 u.c. sample hetero-interface (red triangles) and deeper into the SrTiO_3_ substrate of same sample (green dots). (**c**) Experimental spectrum for LaAlO_3_ away from the interface. Experimental spectra are clearly influenced by cation intermixing rather than extrinsic donors^40^.

At the partial pressure of 10^−4^ Torr used during the PLD growth, no signature of oxygen vacancies could be detected in the O-K edge recorded in the substrate and around the interface by EELS, as observed on Fig. 3. The interfacial O-K EELS spectra reflect intermixing rather than oxygen vacancies^40^,^41^. Although a low level of oxygen non-stoichiometry is not excluded, it would be insufficient to explain the sheet resistance measured and the differences between the 3 and 5 u.c. samples.

Therefore we have focused our work on two mechanisms suspected to play a significant role in the insulator/conductor transition of the hetero-structure: intermixing and structural distortions.

#### A-site intermixing

The investigation of La ↔ Sr intermixing around the interface has been carried out by Medium Energy Ion Spectroscopy (MEIS) with a 100 keV He^+^ beam in the random mode configuration for the 3 and 5 u.c. samples. In this “random” mode, the orientation of the sample was chosen to avoid a channeling of the beam by dense atomic planes and to minimize the probability of a second scattering for the scattered He^+^ on their way out. The energy E of the backscattered He^+^ particles varies with the target element weight, its depth and the scattered angle, θ_sc_. This “random” mode was selected to extract the chemical profiles of Sr and La. Further explanations can be found in the Supplementary Information.

**Figure 4:**
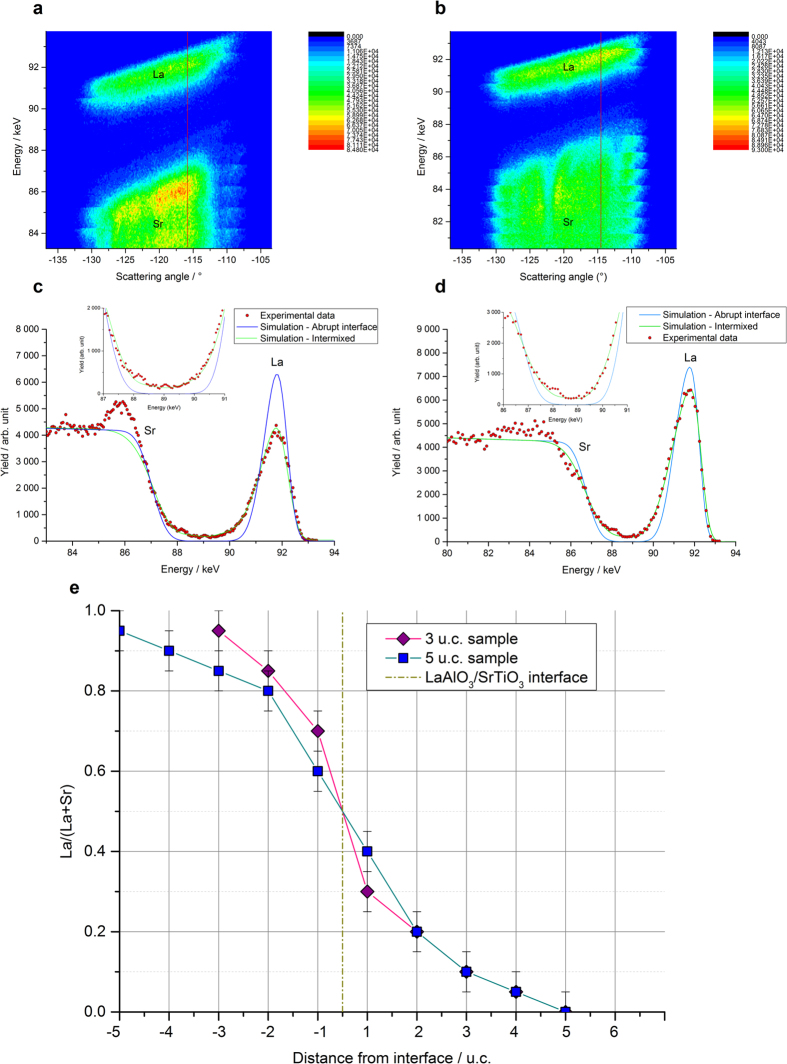
MEIS data in random mode. Incident particles : 100 keV He^+^ ions. (**a**,**b**) MEIS random maps (E_1_, θ_sc_, N) of the samples with a film thicknesses of (**a**) 3 u.c. (**b**) 5 u.c. Top (-bottom) layers of highest (-lowest) energies correspond to He^+^ particles backscattered on La (-Sr) respectively. Red lines locate the position (at a fixed scattering angle) of the profiles extracted. (**c**,**d**) Random MEIS experimental and simulated spectra of the (**c**) 3 u.c. sample with a backscattering angle of 115,4° (**d**) 5 u.c. sample with a backscattering angle of 114,5°. The blue curves simulate MEIS theoretical spectra for non-intermixed and fully stoichiometric LAO/STO heterostructures. Experimental spectra are plotted with red dots, whereas the best simulated hetero-structures are represented by a green curve. (**e**) La/(La+Sr) profile throughout the first atomic layers. The x coordinates locate the cations by the number of unit cells to the interface (+1 = first u.c. in STO, −1 = first u.c. in 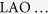). The film contains a cumulative amount of 0.9 +/− 0.05 Sr in the 5 u.c. sample (thus 4.1 +/− 0.05 La) and 0.5 +/− 0.05 Sr in the 3 u.c. one (thus 2.5 +/− 0.05 La). The La counter-diffusion depths were similar for the two samples, with 0.8 La for 5 u.c., and 0.7 La for 3 u.c. films.

Figure 4a,b show the random maps (E, θ_sc_, N) recorded for the 3 and 5 u.c. samples respectively, where N expresses the count number. The N = f(E) curves extracted from these maps at a fixed θ_sc_ are shown in Fig. 4c,d where the red dots correspond to the experimental data. For both samples of 3 u.c. and 5 u.c. thickness, La was distributed over a larger depth than the film thickness and the Sr signal started above the substrate surface clearly exhibiting La ↔ Sr intermixing.

The La ↔ Sr intermixing was quantified by simulations of composition gradients with unit cell resolution. For each layer the ratio x = La/(La + Sr) was optimized to permit the best fit to the experimental curves (see Supplementary Fig. S3). The resulting variations of x across the interface are shown in Fig. 4e. Strontium was found in each layer up to the surface for both films, and the La counter-diffusion depths (≈4–5 u.c) were similar for the two samples. The substitution of La by Sr was slightly larger in the 5 u.c. film than in the 3 u.c. film at any depth, whereas within the substrate, only the first layer contained more La in the 5 u.c. sample than in the 3 u.c. one. The cumulative Sr content in the film was greater than the cumulative La content in the substrate for 5 u.c. film, while the opposite was true for the 3 u.c. film.

**Figure 5:**
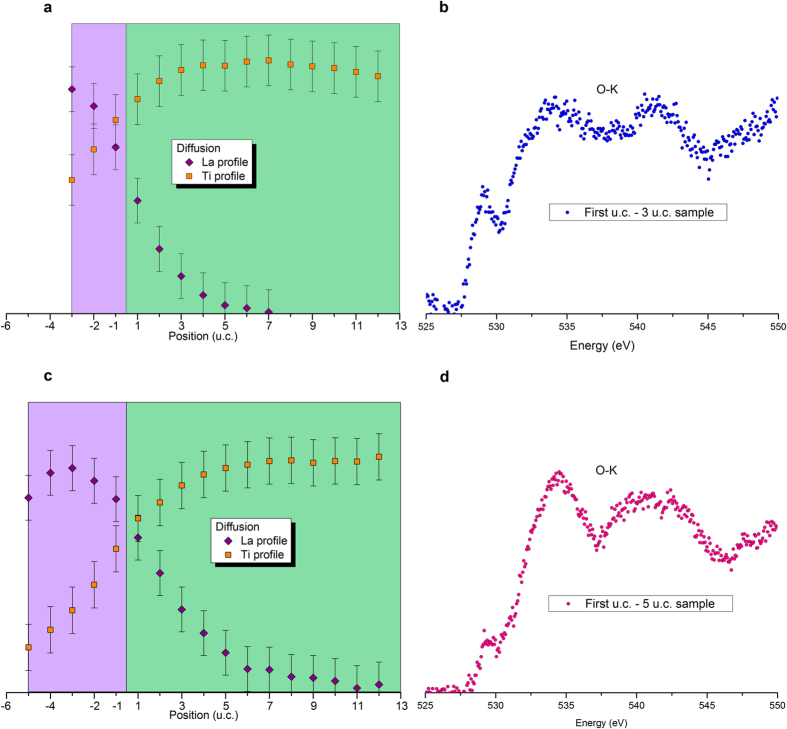
Cationic intermixing A-sites vs B-sites. (**a**–**c**) Diffusion profiles of Ti and La from EELS analyses for the (**a**) 3 u.c. (**c**) 5 u.c. sample. The x coordinates locate the cations by the number of unit cells to the interface (+1 = first u.c. in STO, −1 = first u.c. in 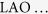). Ti diffused up to the film surface for both samples. Assuming a full occupancy of B-sites, Al would diffuse down to 3/4 u.c. into the substrate of the LAO(3 u.c.)/STO sample, and slightly deeper (4/5 u.c.) into the substrate of the LAO(5 u.c.)/STO sample. La atoms diffused down to about 5 u.c within the substrate of the 3 u.c. sample whereas it was found deeper (6/7 u.c.) into the thicker sample STO. The shallower diffusion depth of B cations compared to A cations results in La_Sr_. donor dopants that are not fully compensated by Al_Ti_’ acceptor dopants and can provide charge carriers. It can be noticed that La cations are detected at deeper depth by EELS than by MEIS. This is explained by the straggling of He^+^ particles that decreases the sensibility of this ion beam analysis with the depth of the analyzed region. (**b**–**d**) EELS fine structure of the O-K edge recorded in the first u.c. under the film surface of the (**b**) 3 u.c. (**d**) 5 u.c. sample. The observed O-K pre-peak, between 529 and 531 eV, is characteristic of the Ti-O π* hybridization^41^ which confirmed the presence of Ti atoms within the very first unit cell of each samples.

The atomically-resolved MEIS depth profiles clearly demonstrated La and Sr intermixing. These exchanges of cations with different oxidation states can stabilize the interface and thus can lead to compensation of the dipole energy which otherwise would form at an ideal, abrupt-interface^22^,^24^,^25^,^26^. A La^3+^ ↔ Sr^2+^ cation exchange around the interface would generate donor-type point defects 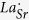 and compensating electrons e’ in the conduction band (CB) of STO. When all cations are considered, intermixing creates a La_x_Sr_1−x_Al_y_Ti_1−y_O_3_ layer, where Al would act as an acceptor dopant. Asymmetry in the intermixing depths on A and B sites occurred as shown experimentally by EELS measurements on Fig. 5, which is consistent with observations in the literature^22^: Al intermixes with Ti at shallower depths and lower ratio than La does with Sr. Therefore x > y implies a net donor doping effect below the interface; however with compensating carrier content (x-y) smaller than the value (x) that would be expected for hypothetical La_x_Sr_1−x_TiO_3_ layer. It has to be mentioned that the deeper diffusion of La measured by EELS (Fig. 5) compared to MEIS (Fig. 4) is explained by a weaker accuracy of MEIS quantification in the deeper layers due to stronger He^+^ straggling.

However one main finding undermines the scenario of a donor-doped under-layer. The difference in the values of x = La/(La + Sr) ratio just below the interface for 5 u.c (x = 0.4 +/− 0.05) and 3 u.c (x = 0.3 +/− 0.05) is not sufficient to explain the discrepancy in terms of electrical properties between these two samples. Therefore correlations of intermixing with the strain levels have been investigated.

#### Local Strain

##### Experimental stain profiles

**Figure 6:**
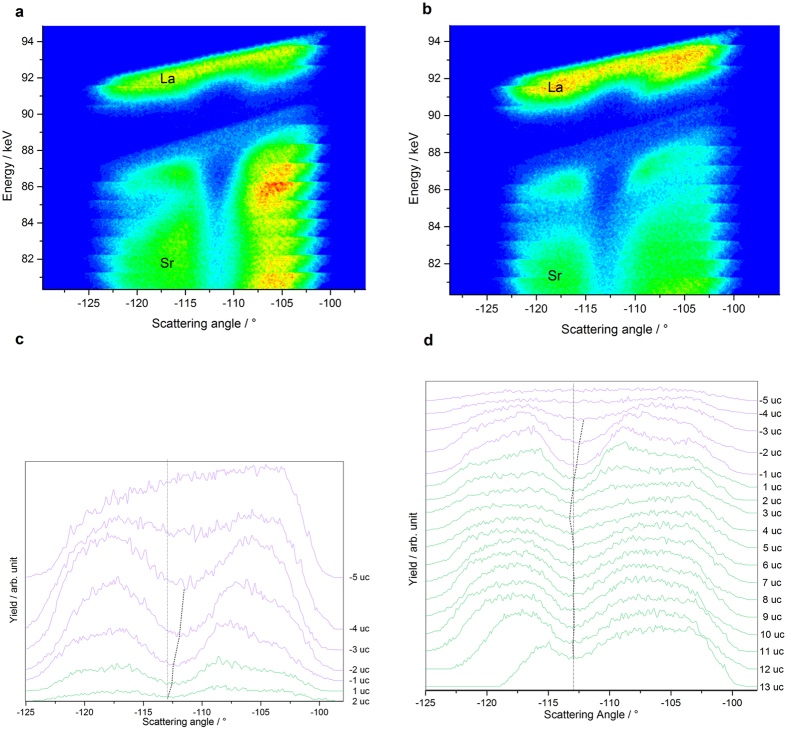
MEIS data in blocking mode. (**a**,**b**) MEIS blocking map (E_1_, θ_sc_, N) of the sample with a film thickness of (**a**) 3 u.c. (**b**) 5 u.c. (**c**,**d**) MEIS [101] blocking dips of the 5 u.c. sample, as a function of depth corresponding to (**c**) La atoms (**d**) Sr atoms located in the film (purple curves) and in the substrate (green curves). Dash-dotted lines are the position of the blocking dips for an unstrained cubic STO (c/a = 1). Dashed lines indicate the position of the blocking dips maxima for each unit cell. In the substrate, deviations of these positions from the dash-dotted line reveal structural distortions.

The strain profiles have been obtained in a blocking mode configuration. In this mode the sample was oriented to maximize a second scattering of the He^+^ particles for a given scattering angle, preventing them to escape the sample. Figure 6a,b present (E, θ_sc_, N) maps for the 3 u.c. and 5 u.c. samples taken in a scattering geometry that promoted blocking in the [101] direction. The He^+^ energy range was selected to analyze both Sr and La scattering centers. A shadowing effect in the distribution of the scattered ions distribution is observed, characteristic of these “blocking dip” patterns. The series of profiles N = f(θ_sc_) at given energies have been extracted to quantify the shift of the maximum dips as a function of energy. Then energies were converted into depth values with unit cell resolution (Further details are in the Supplementary Information). The series of extracted profiles are displayed in Fig. 6c,d for La and Sr. The scattering angle, θ_bk_, inducing the blocking maximum (minimum of intensity) in the [101] direction, was located for each profile by fitting the dip regions with parabola. The variations of θ_bk_, were then translated into variations of c/a ratio. The [101] blocking dips in the substrate at 13 u.c., for the 5 u.c. film, and at 14 u.c., for the 3 u.c. film, from the interface were assumed to belong to an unstrained cubic STO. The corresponding blocking angles were taken as the reference, θ_bk_(ref).

**Figure 7:**
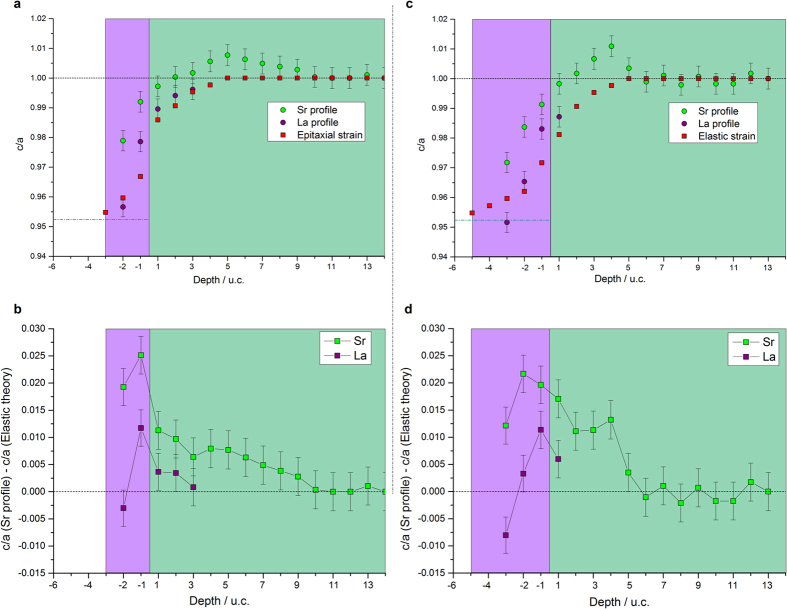
Profiles obtained from MEIS data in blocking mode. The x coordinates locate the cations by the number of unit cells to the interface (+1 = first u.c. in STO, −1 = first u.c. in 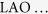). Each data point indicates the distance separating this cation to the A cation located in the above cell. (**a**–**c**) Profiles of c/a for samples with film thicknesses of (**a**) 3 u.c. (**c**) 5 u.c. Purple circles represent the c/a values around La atoms. Green circles represent the c/a values around Sr atoms. Red squares represent the theoretical c/a values for intermixed heterostructure assuming a sole elastic epitaxial strain. Relaxed cell parameters in this intermixed region were determined from their respective chemical profile obtained experimentally by MEIS random mode. The turquoise dash-dotted line indicates the theoretical c/a value for a fully epitaxial growth of LAO, (a_STO_ = a_LAO_) and without intermixing. (**b**–**d**) Difference between the c/a values measured experimentally - from He^+^ scattered from Sr and La atoms - and those calculated from epitaxial strain are plotted for the (**b**) 3 u.c. sample (**d**) 5 u.c. sample.

Figure 7a,c present the variation in c/a for the 3 and 5 u.c. films calculated from θ_bk_ as a function of depth for He^+^ backscattered on La (purple dots) and Sr (green dots). For both samples, the curves could be divided into three regions. The deeper probed parts of the substrate were unstrained (c/a = 1). Then an intermediate region was observed with c/a > 1 in the substrate starting from 9 u.c. for the thinner sample and from 5 u.c. for the thicker one and extending up to 2 u.c. below the interface. The maximum c/a was observed at depths of 5 u.c. and 4 u.c into the substrate for the 3 u.c. and 5 u.c. films, respectively. Finally the region extending from 2 u.c. below the interface up to the film surface exhibited c/a < 1. The differences between the two samples laid mainly in the thicknesses of these regions as will be discussed later.

We note that for a given depth, the c/a ratios calculated from scattering on La and Sr do not match. For blocking in [101] direction, this indicates that the distance between two A-site cations located on the diagonal of the (010) face is affected by intermixing. In the film, the Sr_La_-La_La_ distance is longer than the La_La_-La_La_ one, whereas in the substrate the La_Sr_-Sr_Sr_ distance is shorter than the Sr_Sr_-Sr_Sr_ one. Differences in cation radii as well as electrical charges of the hosted atoms could have induced distortions in the cell as well as a buckling of the layer. Such distortions around the hosted cations have been predicted by modelling (see the following sections).

##### Origins of the strain

In order to investigate further the nature of the strain, we have evaluated the contribution of epitaxial strain to the total strain in these hetero-structures. These epitaxial strains were calculated from a purely elastic model based on unstrained cell parameters deduced, in the intermixed region, from Vegard’s law between STO and LAO (Calculations are described in the Supplementary Information). The strength of this approach is that the intermixing profiles, obtained from MEIS random mode on the same samples, were used for these elastic calculations. The depth variations of c/a, as predicted from epitaxial elastic strain and based on the chemical profile depicted in Fig. 4e are plotted in red squares in Fig. 7a,c. The c/a ratio measured experimentally with MEIS blocking dips around the interface are clearly larger than the values predicted by purely elastic strains. Thus, epitaxial elastic strain cannot be the only factor that explains the strain level measured.

Figure 7b,d plot the differences between the experimental MEIS strain profiles and the theoretical epitaxial strains. The difference reached a maximum around the interface. Several mechanisms can lead to additional strain in such systems such as Jahn-Teller distortions due to the change in valence of Ti cations, ferroelectric-like distortions due to off-centering of cations and other distortions due to ionic defects such as strontium vacancies. The Ti^3+^ concentration was seen to be the highest near the interface. Since the d orbitals, empty for Ti^4+^, are occupied by one electron for Ti^3+^, removal of the degeneracy of the t_2g_ energy levels (d_xy_ , d_xz_, d_yz_ orbitals) is expected. A first scenario would favor a stabilization of the d_xz_ and d_yz_ orbitals due to an elongation of the octahedron of the first STO cell subjected to in-plane compressive stresses from the film. The additional c-lattice expansion measured compared to the strain provided by epitaxial elastic strain could be explained by this Jahn-Teller-like effect^20^,^21^,^22^,^23^. However a second scenario would imply a pure Jahn-Teller effect that stabilizes the d_xy_ orbitals and induces a contraction of the TiO_6_ octahedron^11^. The observed elongation of the cell does not exclude such a contraction of the octahedron, through a buckling of Sr-O-Sr chains^11^, where the oxygen anion planes depart from the cation ones, as was emphasized using first-principles calculations^42^.

Nevertheless the low concentration of Ti^3+^ indicates that other additional origins for this cell distortion must be searched. Ferroelectric-like distortions were also hypothesized to be directly driving the interface electrical characteristics^21^. While they might contribute around the interface, as Ti cations on HAADF images were seen off centered in the 10 u.c. film (Fig. 1a), they cannot explain the distortions seen deeper in the substrate.

A third factor that can influence the lattice strain is the presence of cation vacancies. Owing to their high formation energies, titanium vacancies 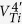 will not be considered^43^,^44^. Strontium vacancy formation is coupled to the creation of oxygen vacancies via Schottky reactions, Frenkel pairs being energetically less favorable^45^. 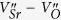 defect complexes could be formed during the processing of the STO substrate, after the melt-growth, upon cooling the ingot. 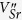 concentration in the substrate could either exceed their equilibrium concentration at room temperature due to their low diffusion rates, 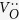 being more mobile, or can be annealed to near equilibrium concentrations. Strontium vacancies could also be formed on the substrate surface sublayer due to Sr-La intermixing. At the oxygen partial pressure used, the La donors are partially compensated by 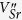 (ionic compensation)^46^, the film being a sink for the expelled Sr ions. The lower level of electron compensation at such oxygen partial pressure could explain the high valence measured for Ti cations. Freedman *et al*.^44^ found that a strontium vacancy induces an overall expansive strain although the nearby relaxation may differ. As in-plane parameters are more constrained, this may result in an expansion of the cell dimension in the growth direction^39^,^47^. The reduction of the 2D charge confinement along the c-axis at LAO/STO interface when the substrate is subjected to in-plane compression has been reported^48^,^49^,^50^, and explained by a dilution of the interfacial charge carrier density, with the mobile charges transferred deeper in the STO substrate.

### Modeling of the structural relaxation around hetero-interface by first-principles calculations

#### Structural relaxation around abrupt interfaces

**Figure 8:**
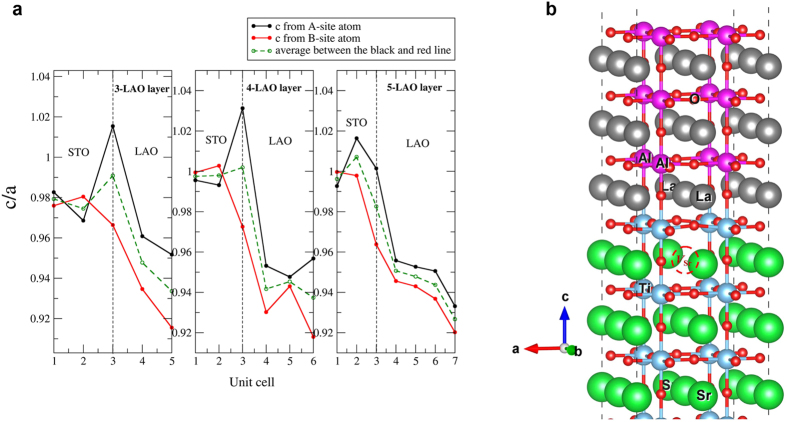
Modeling results. (**a**) Calculated c/a in non-intermixed layers of LAO on a 5 layer STO calculated in a symmetric slab geometry. (The x coordinate “3” for the black curve indicates the La-Sr distance across the interface and corresponds to the x coordinate “1” in Fig. 7). (**b**) Relaxation around a strontium vacancy located in the first u.c. under the interface. The 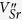 is indicated by a dashed circle. One can see the buckling of the layer above it and the O moving away from it. One can also see the La and Sr directly above the 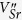 to move toward it. The Ti-Ti distances in the c-direction surrounding the vacancy containing layer is 0.1 Å smaller than in the layer below it without vacancy.

For comparison with the measured strain profiles, we first calculated the structural relaxation for perfect non intermixed structures as reference. Figure 8a reports the c/a ratio in each layer. Here “a” is the common in-plane lattice constant throughout the cell which is set by the unstrained STO lattice constant. The “c” for each unit cell layer is determined either from the A-cation (La or Sr) distance perpendicular to the interface (black curve, to be compared with La or Sr profiles of Fig. 7) or the B-cation (Al or Ti) distance perpendicular to the interface (red curve) or from the average of these A-A and B-B distances (dashed green line). Within linear elastic theory, as explained in the Supplementary Information, the c/a ratio can be calculated for a pure non-intermixed LAO pseudomorphically (biaxially) strained on STO. This predicts a c/a = 0.95, (see blue dashed line in the film region of Fig. 7a,c) which one can see in Fig. 8a is close to the value obtained from the first-principles calculation in the middle of the LAO layer. In agreement with Pentcheva *et al*.^51^, we find a buckling of the layers, with the oxygens moving toward the surface relative to the cations in each layer. However, we see that at the free surface layer the local c/a is significantly smaller (0.93 on the average curve) because of surface relaxation that induced a significant decrease of the buckling for top surface AlO_2_ layer. Details can be found in Fongkaew *et al*.^42^. The c/a calculated from elastic theory, shown as red squares in Fig. 7, assumes that the local lattice constant is purely determined by Vegard’s law based on the degree of intermixing experimentally measured in each layer but does not include this relaxation effect. Adding this effect would make the slope slightly stronger downward toward the surface and would improve the agreement with experiment slightly.

We now address the discussion of the c/a overshoot in the STO region near the interface compared to the elastic model. The calculations suggest that the c/a measured as distance between A cations (black curve in Fig. 8a) decreases from the interface toward the STO. This occurs for both 3 u.c. and 5 u.c. heterostructures, although the highest c/a occurs right at the interface for the 3 u.c. (or 4 u.c.) case and one layer deeper in the STO in the 5 u.c. case. This difference is related to distinct buckling modes. In the 3 u.c. case, the Sr move away from the interface relative to the O in the same layer, whereas in the 5 u.c. case, they move toward the interface. Our modelling shows that, in addition to the variation of the c/a due to intermixing which is modeled by the elasticity theory in Fig. 7 (red squares), there is a trend due to the relaxation of the layers which helps to explain the increased c/a near the interface. Our calculations unfortunately do not allow us to gauge in much detail how this varies as function of distance into the STO for different cases because the thickness of the STO in our model calculation is too small to allow for such an analysis.

#### Structural relaxation around intermixed interfaces

In order to approach the intermixed case, we have studied the behavior near a Ti and a Sr on the LAO side^42^. On one hand, if we swap a Ti and Al across the interface, both move farther away from the interface, as shown in Figures 10 and 11 of Fongkaew *et al*.^42^. This means the Ti-Ti distance and hence c/a measured locally from B cations across the interface would increase while the Ti-Al with the next AlO_2_ layer toward the surface would decrease. This could explain the trend of the Fig. 7b,d curves departing from linear elasticity theory.

On the other hand, if we replace Sr by La in a layer near the interface in STO and vice versa, we find that the buckling of the layer is reduced for the swapped atoms. In the film, Sr moves out less toward the surface than La. In other words, relative to La, Sr is closer to the interface. This means that the c/a measured from the Sr atoms in the film would be larger than from the La atoms. This is in agreement with the measured MEIS profiles (Fig. 7) showing a larger interlayer distance measured from Sr_La_ blocking curves than from La_La_ ones. The effects of these various intermixings on the charge density accumulated at the interface in the 2DEG, the potential profiles and electronic structure are discussed by Fongkaew *et al*.^42^.

#### Structural relaxation near a strontium vacancy

The relaxed structure near a Sr vacancy at the interface has also been modeled. The main result of this modeling is that near the Sr vacancy there is an outward relaxation of the oxygens mainly in interface TiO_2_ layer above it. The Ti-Ti distance in the c-direction for the layer without 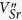 is 3.77 Å, while in the layer with 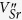 it reaches 3.967 Å. Both these tendencies agree with Freedman *et al*.^44^ who have studied the long-range and short-range strain distortions caused by Sr-vacancies using a shell force-constant model including electrostatic effects. Our calculations however show a local decreasing of c/a (as measured from either the A or B atoms) rather than a crystal expansion as indicated by Freedman’s calculations. However our model does not allow us to reliably determine the long-range strain effect because of the high concentration of 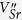 in the model. In Fig. 8b we can clearly see the O above the 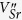 moving away from the vacancy, and one can also see the La and Sr directly above and below it to move toward the vacancy. This local distortion may be compensated by a long-range overall expansive strain (as indicated by Freedman’s calculations).

### Conducting versus insulating interfaces

It has been shown that the levels of intermixing and titanium reduction did not differ sufficiently between the 3 and 5 u.c. samples to explain the large discrepancies measured in their electrical conductivities. The main difference lies in the strain field developed into the two STO substrates. Figure 7b,d show that, if regions with c/a > 1 extends deeper in the substrate of the 3 u.c. sample, the area enclosed between c/a > 1 and c/a = 1 in the substrates are similar for the two samples. This indicates a difference in the distributions of the Sr vacancies more than in their concentrations. The lower dipolar field built in the thinner film induces a weaker 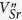 attraction toward the interface than in the 5 u.c. sample, explaining the deeper region with c/a > 1 in the 3 u.c. assembly. This in turn would increase the dilution of the charge carrier and reduce the conductivity with respect to the 5 u.c. sample.

In the conductive samples the density of electrons transferred to the interface is not sufficient to cancel the electrical field, and structural distortions or point defects are still present to screen the remaining field. The in-plane compression strains observed are reported to reduce the concentration and mobility of the charge carriers. The point defects are known to act as trapping or scattering centers.

This study suggests a competition between electronic compensation and donor doping on the one hand, and polar distortions and point defects on the other hand, to balance the electrostatic field formed due to polarization discontinuity in the polar film, the structural relaxation playing a crucial role on the electronic conduction of such heterostructures.

## Conclusion

MEIS was used to correlate the intermixing and the structural distortions with an atomic depth resolution within LAO/STO heterostructures above and below the critical thickness for insulator/conductor transition. In addition, the oxidation state of Ti atoms and the O vacancies were characterized by EELS. Neither electronic reconstruction nor anionic vacancies alone can explain the carrier density observed. Intermixing is demonstrated in the two samples, excluding a donor doping scenario as single mechanism. The measured c/a ratio are larger than those predicted by epitaxial strains obtained from an elastic calculation taking intermixing into account. This indicates that compressive electrostatic forces developed around the interface, and extended deeper into the substrate in the 3 u.c. sample, reducing the confinement and diluting the interfacial charge carrier. A complex competition between donor doping, structural distortions and reconstruction, and ionic compensation is revealed. This paper highlights the complexity of the scenario occurring at the interface responsible for the conductivity in LAO/STO heterostructures.

## Methods

### Film Growth

LaAlO_3_ (LAO) films on SrTiO_3_ (STO) (001) substrates were grown by Pulsed-Laser Deposition (PLD). Prior to the film growth, the SrTiO_3_ (STO) substrates were etched with a chemical solution of ammonium fluoride and hydrofluoric acid at pH = 6 to obtain a TiO_2_-terminated surface and then annealed at 950 °C for one hour in an oxygen-rich atmosphere. The surface morphology was checked with AFM (Agilent Technologies). In the PLD chamber the base pressure of the chamber was 10^−6^ Torr and was increased to an O_2_ partial pressure of 10^−4^ Torr via an MKS Mass Flow Controller and Cold Cathode. The growth was performed at a temperature of about 750 °C with an initial ramping rate of about 10 °C/min up to 500 °C and then about 30 °C/min up to the deposition temperature. The LAO target was ablated using a 248 nm KrF excimer (Coherent Inc.) laser with a fluence of about 1.2 J/cm^2^ and a repetition rate of 2 Hz. LAO films were grown at a rate of 15 pulses per layer and the growth rate was followed *in situ* by oscillations in Reflection High-Energy Electron Diffraction (RHEED) patterns (STAIB Instruments). After deposition, films were brought to room temperature at cooling rates of about 10 °C/min then about 5 °C/min.

Following this protocol, films 3 and 5 unit cells (u.c.) thick were grown on TiO_2_-terminated STO substrates to optimize the resolution in MEIS, compared to thicker films.

### Scanning Transmission Electron Microscopy and Electron Energy-Loss spectroscopy

The growth epitaxy and interface coherency in the experimental conditions used for PLD were analyzed on both samples by Scanning Transmission Electron Microscopy (STEM) using a Nion UltraSTEM 200 operating at 100 kV and a High-Angle Annular Dark-Field (HAADF) detector with an inner collection angle of 70 mrad. The microscope was equipped with a spherical aberration corrector, which enabled a probe-size of under 0.1 nm to be obtained. EELS spectra were acquired with a Gatan Enfina spectrometer and a custom-made EELS camera. The energy resolution attained for this set of experiments was 0.5 eV.

The O-K, Ti-L_2,3_, and La-M_4,5_ absorption edges were recorded to probe the oxygen vacancy concentration throughout the sample, titanium oxidation state around the LaAlO_3_/SrTiO_3_ hetero-interface, and the diffusion of A-site cations vs B-site cations.

### Electrical characterization

Electrical transport measurements were made in a physical property measurement system (PPMS, Quantum Design Inc.) with temperature varying from 2 K to room temperature. Sheet resistance, carrier density and Hall mobility were obtained by Hall effect measurements in a four-probe Van der Pauw configuration. The electrical contacts to LAO/STO interface were achieved by direct wirebonding of aluminum wire to the sample. After confirming the Ohmic behavior of contacts, four-probe sheet resistance and Hall resistance of 5 u.c. sample were measured using standard lock-in technique with less than 1 mV excitation at 13 Hz. For the insulating 3 u.c. sample, lock-in measurement of resistance was not possible. D.C. current-voltage measurements indicated a resistance higher than >100 MΩ.

### MEIS

Medium Energy Ion Spectroscopy (MEIS) was performed to investigate the chemical and strain profiles of the LAO/STO heterostructures near the samples surface, using a He^+^ collimated beam of 100 keV hitting the film surface at a given incident angle relative to the crystal cell directions. The energy E and angle θ_sc_ of the scattered He^+^ that escape the solid are analyzed simultaneously using a toroidal electrostatic analyzer with an energy resolution ∆E/E = 3 × 10^−3^ and an angular resolution of 0.1°. The incident angle was carefully chosen to induce two specific scattering geometries, the random and blocking modes. Further details can be found in the Supplementary Information.

### Modeling

First-principles calculations were performed within the density functional theory using PBE exchange correlation generalized gradient approximation^52^,^53^ and projector augmented wave potentials^54^,^55^ in the VASP code^56^,^57^. The supercells were set up in a symmetric way with 5.5 (001) oriented STO layers and either 3, 4, or 5 unit cell LAO layers on either side, followed by a sufficiently thick vacuum region. The systems were fully relaxed until all forces are smaller than 0.02 meV/Å and the plane wave cut-off used was 500 eV. For k-space integration, the Monkhorst-Pack scheme with 7 × 7 × 1 k-point sampling was employed.

## Additional Information

**How to cite this article**: Zaid, H. *et al*. Atomic-resolved depth profile of strain and cation intermixing around LaAlO_3_ /SrTiO_3_ interfaces. *Sci. Rep*. **6**, 28118; doi: 10.1038/srep28118 (2016).

## Supplementary Material

Supplementary Information
