# Supplementary material for: Atomic-resolved depth profile of strain and cation intermixing around LaAlO3/SrTiO3 interfaces
Source: Sci Rep. 2016 Jun 15;6:28118. doi: 10.1038/srep28118 (PMC4908387; doi:10.1038/srep28118)
Supplement: Supplementary Information [file srep28118-s1.pdf]

Supplementary Information

Atomic-resolved depth profile of strain and cation intermixing around $LaAlO_3/SrTiO_3$ interfaces.

H. Zaid[1*], M.H. Berger[1], D. Jalabert[2,3], M. Walls[4], R. Akrobetu[5], I. Fongkaew[6], W. R. L. Lambrecht[6], N. J. Goble[6], X. P. A. Gao[6], P. Berger[7], A. Sehirlioglu[5]

[1] MINES ParisTech, PSL Research University, MAT - Centre des matériaux, CNRS UMR 7633, BP 87 91003 Evry, France

[2] Université Grenoble Alpes, INAC-SP2M, LEMMA, F-38000 Grenoble, France

[3] CEA, INAC-SP2M, LEMMA, F-38000 Grenoble, France

[4] Laboratoire de Physique des Solides, Université Paris Sud, Bât 510, 91405 Orsay, France

[5] Departments of Materials Science and Engineering, Case Western Reserve University, Cleveland, Ohio, USA

[6] Departments of Physics, Case Western Reserve University, Cleveland, Ohio, USA

[7] NIMBE, CEA, CNRS, Université Paris-Saclay, CEA Saclay 91191 Gif sur Yvette Cedex

[*] email : hicham.zaid@mines-paristech.fr

Supplementary Methods

Medium energy ion scattering

Medium-Energy Ion Scattering (MEIS) is based on the same physical principle as Rutherford Backscattering Spectroscopy (RBS) analyzing the interactions between a charged particle and a solid. The difference lies in the range of energy used, a few hundred keV, for the incident beam in MEIS compared to several MeV for RBS. This gives the particles a high stopping power in MEIS, allowing the analysis with unit cell resolution of the near-surface atomic layers.

The energy E_{sc} of the particles backscattered by surface atoms can be predicted knowing the kinematic factor k given by

$$k = \frac{E_{sc}}{E_0} = \left[\frac{\sqrt{M^2 - m^2 \sin^2 \theta_{sc}} + m\, cos\theta_{sc}}{M + m}\right]^2 = k(M, m, \theta_{sc})$$

With E_0 the energy of the incident He^+ particles, m their mass, M the target atom mass, θ_{sc} the scattering angle. For particles backscattered by atoms at a distance *z* under the surface, an additional energy loss is introduced resulting from the interaction of the particles with the solid along their in- and out-going paths. This additional energy loss is calculated from the stopping power of He^+ in the target material[1] and the couple (θ_i, θ_{sc}) defining the lengths of the in- and out- paths. These data on energy loss can provide, at a given depth in the sample, the average kinetic energy of the ions. However, the statistical processes involved in slowing down lead to energy loss fluctuations, commonly called energy straggling. In the medium energy range, these fluctuations dominate the depth resolution. Details of the theory can be found in the referred study[2]. This analysis shows that the energy of ions scattered at a defined depth z and collected by the electrostatic analyzer is dependent on the scattering angle θ_{sc} through two physical mechanisms, the scattering event (interaction with nuclei) and the stopping power (mainly interaction with the electron cloud).

Scattering geometry for MEIS analysis

The standard method using the medium energy ion scattering (MEIS) technique for structural analysis consists of aligning a crystallographic direction of the sample on the angle-resolved detector entrance and scanning the energy of the scattered ions with an electrostatic energy analyzer. Supplementary Figure S4 schematizes the three degrees of freedom (θ, ψ, ϕ) for angular orientation of the sample fixed on the goniometer. The beam hits the sample with an angle θ_i with respect to the surface normal ([001] direction). The scattered particles escape the sample with angles θ_{sc} as schematized in this figure and are recorded by the detector. This figure illustrates a first map recorded in order to choose the scattering geometries corresponding to the random and blocking modes. When a beam of light ions impinges a single-crystal sample, the scattered ion distribution observed around this sample presents minima aligned with the crystalline directions. In these particular directions, the scattered ions are deflected from their initial trajectories by the nuclei of the upper layers and one thus observes shadowing effects in the distribution of scattered ions. In this example, the energy of scattered He^+ collected was fixed at 85.77 eV, which corresponds, for the range of θ_{sc} chosen, to He^+ ions scattered on Sr atoms located in the substrate. The angles θ and ψ were fixed at -26° and 2.5° respectively (θ_i=26.1°). Then the sample was tilted around its [001] axis from ϕ=-60° to +60°. For each tilt angle, the count number N was recorded as a function of θ_{sc} in the interval [-113°, -87°]. This map (ϕ, θ_{sc} , N) encloses large horizontal darker strips at given tilt angles ϕ, the strong decrease in the signal is caused by the channeling of the incident beam by the crystal planes for these tilt angles, which dramatically reduces the scattering probability. The thinner horizontal or curved and tilted lines correspond to blocking planes due to scattered particles deflected from their initial trajectories by the nuclei of the first upper layers. The intersections of such blocking lines (planes) correspond to blocking directions (“holes” in the map). The map exhibits the symmetry of the cubic crystal and can be indexed in a similar way to a stereographic projection[3]. The tilt angle ϕ and scattering angle θ_{sc} for the [101] blocking direction were more precisely determined on a second (ϕ, θ_{sc}) map built with smaller steps for the tilt angle scan (see

Supplementary Figure S4). With these maps, the scattering geometry (θ, ψ, ϕ) and range of θ_{sc} for the two modes, random and blocking by [101], could be chosen. The electrostatic analyzer was tuned to collect He^+ particles with energy that corresponded to ions backscattered by lanthanum and strontium atoms located between the film surface and the plane situated at around 15 u.c. under the interface. The measurements were focused on this energy range in order to optimize the energy resolution. The investigation of the Al ↔ Ti intermixing was not possible as the Al signal was buried into the Ti plateau, making reliable quantification extremely difficult.

MEIS experimental chemical spectra

The experimental MEIS chemical profiles have been simulated using an in-house software similar to commercially available programs (for instance SIMNRA[4]) but with specific parameters more adapted to the medium energy range used. As an example, the screening of the nuclei charge by the surrounding electrons modeled with the L'Ecuyer approximation[5] and the energy straggling is based on the calculations of Chu[6]. Moreover we made the assumption of full occupancy in the ABO_3 perovskites by La or Sr on the A sites and Al or Ti on the B sites. Therefore the compositions ascribed to the layers were $(La_xSr_{1-x})(Al_yTi_{1-y})O_3$. The y values were set to 1 in the film and 0 in the substrate. It must be noted that equalizing y to x (same amount of intermixing on A and B sites) did not induce detectable variations in the fit of the Sr and La signals.

Blocking mode configuration

The MEIS blocking mode consists of sending He^+ particles in a direction that promotes additional scattering by atoms situated one layer above the first backscattering center (see Supplementary Figure S5). This involves fewer particles recorded by the detector and it implies a shadowing effect intimately linked with the position of the second scattering atom. Moreover, the kinetic energy of the scattered ions can be related to the mass and hence atomic number of the target atom and to its depth via a knowledge of the kinematic factor formula and the corresponding stopping power of the

ions in the sample, allowing one to measure the strain depth profile by fixing a selected geometry and scanning the energy of the scattered ions[3].

To convert energy into depth values, it was assumed that He^+ particles backscattered on atoms M at the same depth with distinct scattering angles θ_{sc1} and θ_{sc2} , will escape the material with energies E_1 and E_2 with $E_1/E_2 = k_1(\theta_{sc1})/k_2(\theta_{sc2})$. This assumes that the difference ($E_{1out} - E_{2out}$) between the energies lost by the particles on their way out at θ_{sc1} (path length L_1, energy lost E_{1out}) and θ_{sc2} (path length L_2, energy lost E_{2out}) could be neglected. The stopping power over a distance (L_1-L_2) was assumed to introduce only a second order effect.

The strain profiles of this study were obtained in a geometry favoring the [101] blocking dips. This second scattering reduces the number of particles reaching the detector in this angular direction, denoted θ_{bk}, thereby producing a minimum of intensity in the (E_1, θ_{scat}, N) maps for $\theta_{sc} = \theta_{bk}$. The variations in θ_{bk} with E_1 for blocking along [101] give access to the depth variations in the cell parameter ratio c/a. The [101] blocking angle in the cubic STO far from the interface was taken as a reference, θ_{bk}(ref) , for c/a =1, as schematized in (see Supplementary Figure S5). The variation in c/a at a depth z was then derived from θ_{bk} (z) via the following equation:

$$\frac{c}{a}(z) = \tan[45 - (\theta_{bk}(z) - \theta_{bk}(ref))]$$

Therefore if θ_{bk}(z) differs from θ_{bk} (ref), it means that the structure is no longer cubic. Higher θ_{bk}(z) (absolute value) corresponds to c/a(z) higher than unity. As well as, lower θ_{bk}(z) (absolute value) corresponds to c/a(z) lower than unity.

Epitaxial strain determination

In order to determine the different origins of cell distortion in our LAO/STO heterostructures, we have subtracted to the total deformation the part assigned to epitaxial strain, taking into account the intermixing determined from our MEIS chemical profiles. This direct coupling of strain and chemical profile by MEIS in oxide heterostrucutre is the most innovative approach of this work.

For this analysis, the in-plane cell parameters for LAO and STO were fixed to that of cubic STO (0.3905 nm). This assumption is justified by a previously published work, referenced[7] in the main body of the text, showing that the in-plane cell parameter of a 4.9 nm thick LAO film was 0.39028 nm (0.06% difference from that of STO) as measured by reciprocal lattice mapping (RLM).

The theoretical local epitaxial strain was calculated for the exact composition of each layer determined from the experimental chemical profiles. The cell parameter of each fully relaxed $La_xSr_{1-x}Al_xTi_{1-x}O_3$ slices was calculated based on Vegard's law:

$$a_0(x) = (1-x)\cdot a_{STO} + x\cdot a_{LAO}$$

The in-plane stress deforms the cubic cell into a quadratic cell with parameters $a_{//} = a_{STO}$ and $a_{\perp} = c$. The corresponding in-plain $\varepsilon_{//}$ and out of plane $\varepsilon_{\perp}$ elastic strains are related through the equation below:

$$\frac{\varepsilon_{//}}{\varepsilon\perp} = \frac{a_{STO}-a_0}{a_0}\cdot\frac{a_0}{c-a_0} = -\frac{(1-\nu)}{2\nu}$$

With ν the Poisson ratio of $La_xSr_{1-x}Al_xTi_{1-x}O_3$ estimated from a mixing rule between $\nu_{LAO} = 0.24$[8], and $\nu_{STO} = 0.23$[9]. This equation can be reformulated into:

$$\frac{c}{a_{STO}} = -\frac{2\nu}{1-\nu} + \frac{1+\nu}{1-\nu}\cdot\frac{a_0(x)}{a_{STO}}$$

The as-calculated ratios c/a_{STO} , deduced from the epitaxial strain, have been subtracted to the total c/a ratio determined from MEIS blocking mode maps.

Supplementary Figures

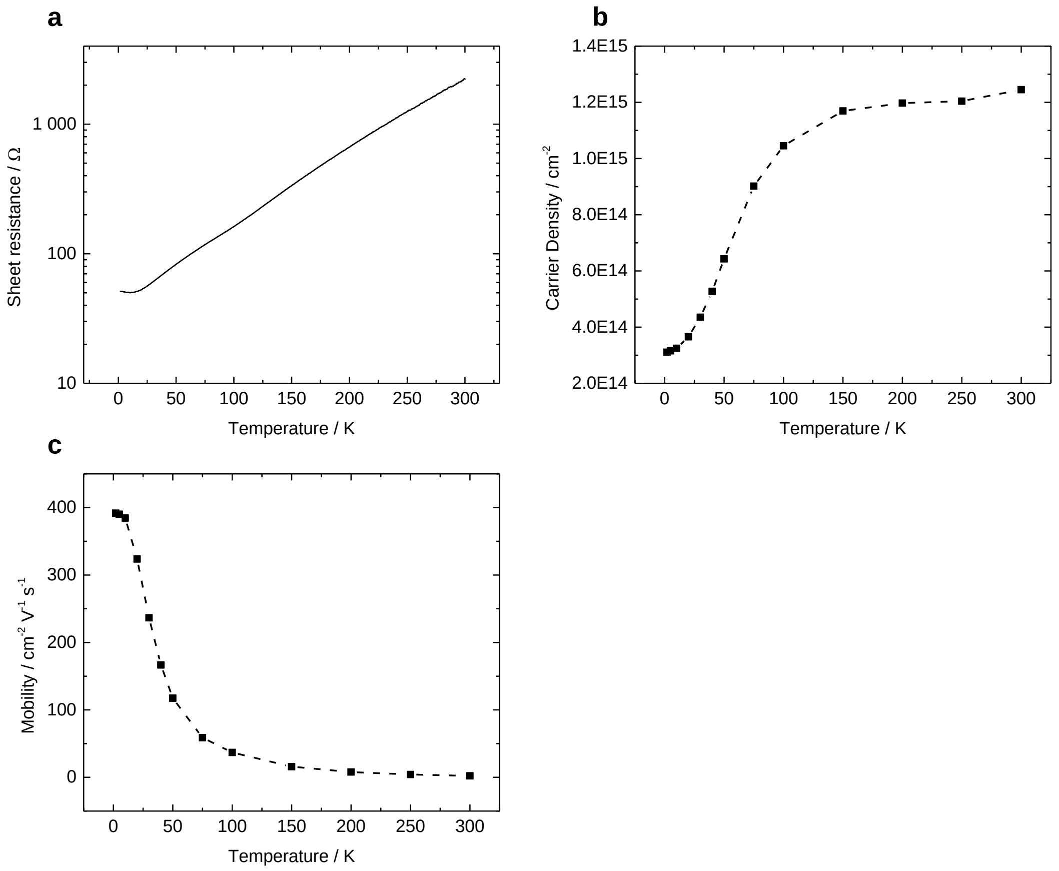

Supplementary Figure S1 | Electrical measurements of the sample with a film thickness of 5 u.c.. Evolution of (**a**) sheet resistance, (**b**) carrier density, (**c**) Hall mobility, as a function of temperature. The sample exhibits metallic conductivity with a carrier density larger than 1.2×10^{15} cm^{-2} at room temperature. The sample with film thickness of 3 u.c. was too insulating to be measured by lock-in measurement of resistance and DC current-voltage measurements indicated a resistance higher than 100 MΩ.

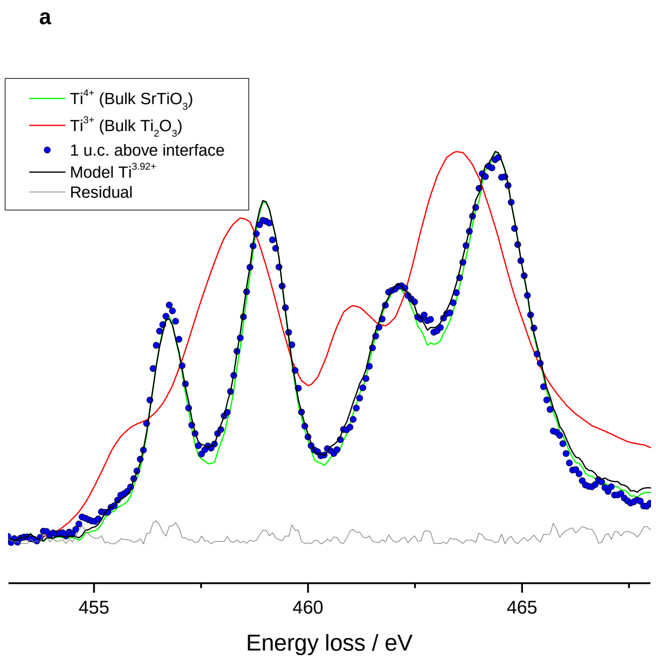

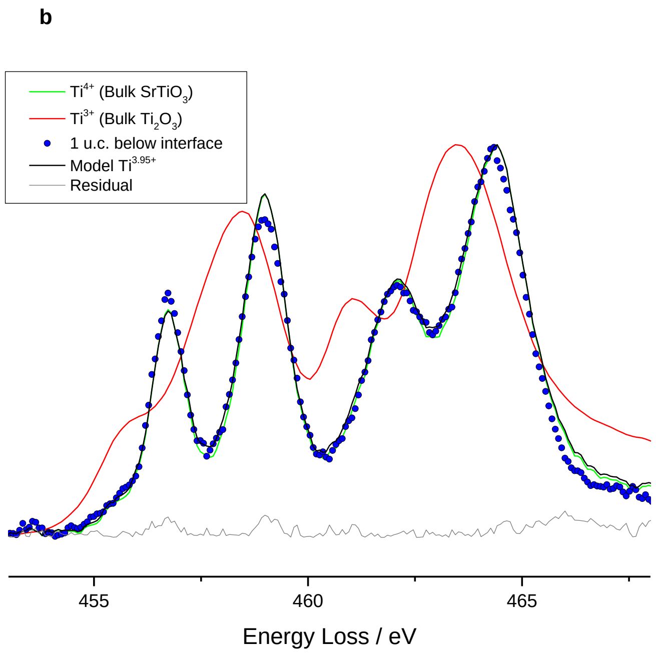

Supplementary Figure S2 | Ti-$L_{2,3}$ edge EELS taken near the $LaAlO_3/SrTiO_3$ interface. EELS spectrum at the unit cell (**a**) above (**b**) under the interface of 5 u.c. sample (Blue curve). The contributions of Ti^{4+} and Ti^{3+} to the Ti edge were deduced from a linear combination of two reference spectra for Ti^{4+} ($SrTiO_3$ away from the interface - green curve) and Ti^{3+} (Bulk Ti_2O_3 - red curve) recorded on the same spectrometer. The method of least squares has been used to fit the experimental data to the simulated spectra. Taking into account charge carriers located in the unit cell under, above and at the

interface, results in a carrier density lower than $1.5x10^{14}$ cm^{-2}. This indicates that the conduction measured in the 5 u.c. sample is not confined to two dimensions.

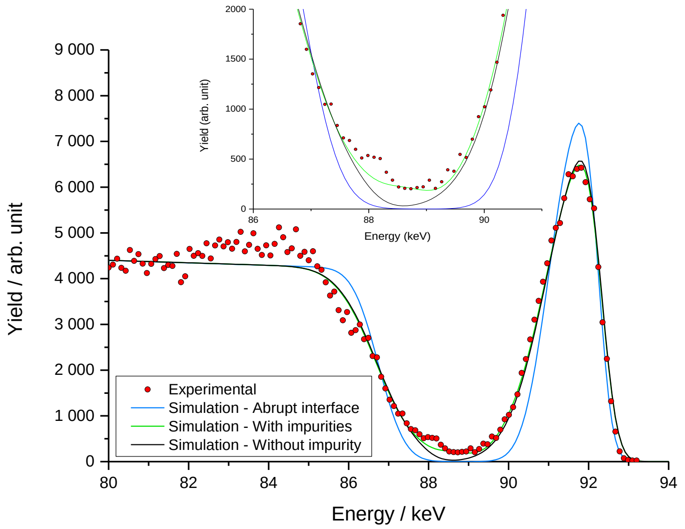

Supplementary Figure S3 | 100 keV He^+ Random MEIS experimental spectrum (red dots) collected with a backscattering angle of 114,5° and simulated spectra (continuous curves) from LAO(5 u.c.)/STO system. The blue curve simulates an ideal stoichiometric heterostructure with an abrupt interface. The black and green curves were obtained by simulating the heterostructure with stacks of 1 u.c. slices of distinct La and Sr compositions. The stacks used for these two curves differ from each other only by the impurity concentrations introduced. The black curve is a simulation without impurity, which does not fit the signal recorded between 55 and 89 keV. The fit is improved when less than 1 at % of Mo, Nb or Ag are added inside the sample (green curve). The first two elements could come from the Inconel disk supporting the STO substrate in the PLD chamber and the last one from the silver paint used to fix the substrate. Other artifacts, related to the use of an energy analyzer, could contribute to the signal in this energy range such as multiple ion scatterings or photon emission. The La/Sr ratios introduced in the simulated stacks have been plotted in Figure 4.

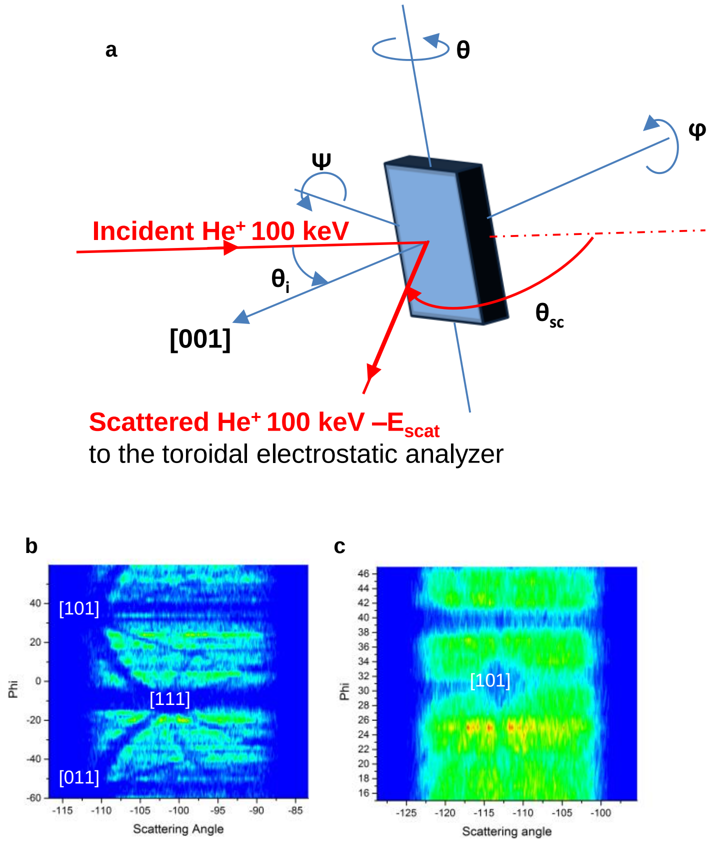

Supplementary Figure S4 | Scattering configuration for MEIS analysis. **a**, Definition of the angles used to orientate the sample fixed on the MEIS goniometer. **b**, Typical MEIS cartography (ϕ, θ_{sc}, N) recorded at a fixed energy corresponding to the substrate region and for a large range of ϕ; N is the count number. Large horizontal stripes correspond to channeling planes, finer horizontal or curved stripes to blocking planes. The orientation of the incident and scattered beams with respect to the STO crystal will be chosen from such maps, to favor blocking or random directions. **c**, Similar MEIS map (ϕ , θ_{sc}, N) using a shorter range of ϕ to localize more precisely the [101] blocking direction.

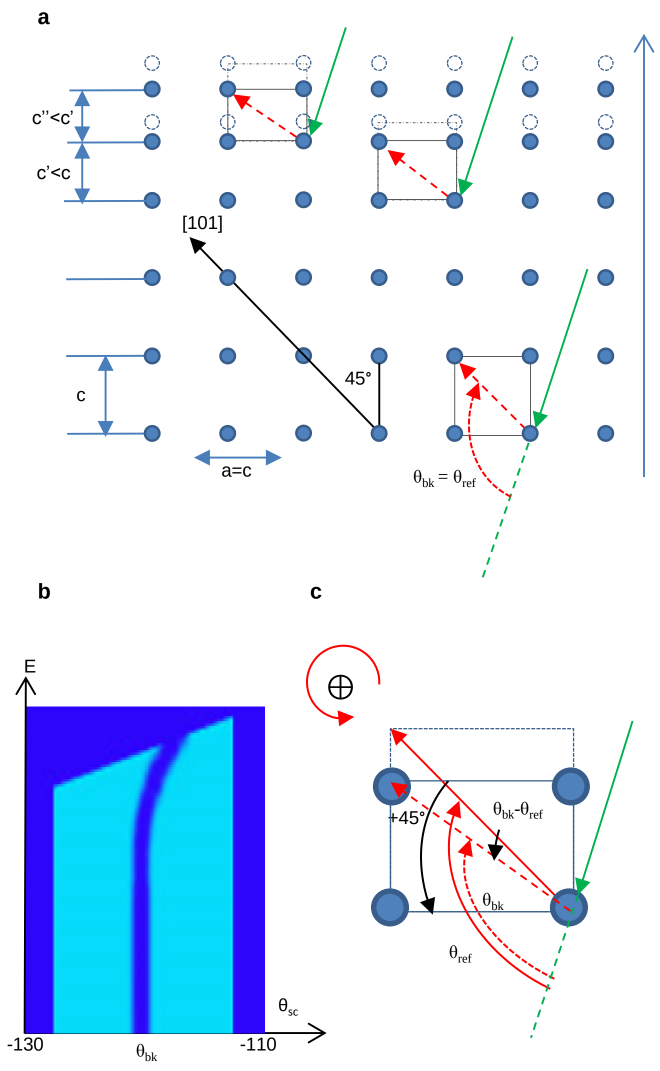

Supplementary Figure S5 | MEIS Blocking mode configuration. **a**, Blocking occurs when a He^+ particle scattered by a nucleus of the sample meets another nucleus before arising from the sample surface, and is deflected from its trajectory by the Coulomb interaction. The angular configuration chosen favors blocking of particles backscattered in the [101] direction. The blocking is proportional to Rutherford cross section and the width of the shadow cone is proportional the inverse of the square root of the atomic distance between the two atoms. Therefore the observed reduction in the Sr or La scattering yield at an angle θ_{bk} is caused by a second scattering on A site cations situated on the layer just above the first scattering center in the [101] direction. **b**, Schematic map (E, θ_{sc}, N) with a tilt of the "blocking dip" angles towards lower absolute angles for higher He^+ energies. This reveals a reduction of the c/a parameter as the distance toward the surface decreases. **c**, Schematic drawing

of the geometry allowing to link blocking angle and cell geometry. According to the [101] direction, the blocking occurs for an angle of 45° in a cubic structure, and depart from this angle for $c/a \neq 1$.

Supplementary References

1. Ziegler, J. F. *Helium: Stopping Powers and Ranges in All Elemental Matter*. **4,** (Pergamon Press, 1977).
2. Chu, W.-K., Mayer, J. W. & Nicolet, M.-A. *Backscattering Spectrometry*. (Academic Press, 1978).
3. Jalabert, D. Real space structural analysis using 3D MEIS spectra from a toroidal electrostatic analyzer with 2D detector. *Nucl. Instrum. Methods Phys. Res. Sect. B Beam Interact. Mater. At.* **270,** 19–22 (2012).
4. Mayer, M. SIMNRA, a simulation program for the analysis of NRA, RBS and ERDA. in *AIP Conference Proceedings* **475,** 541–544 (AIP Publishing, 1999).
5. L'Ecuyer, J., Davies, J. A. & Matsunami, N. How accurate are absolute rutherford backscattering yields. *Nucl. Instrum. Methods* **160,** 337–346 (1979).
6. Chu, W. K. Calculation of energy straggling for protons and helium ions. *Phys. Rev. A* **13,** 2057–2060 (1976).
7. Wei, W. & Sehirlioglu, A. Strain relaxation analysis of LaAlO3/SrTiO3 heterostructure using reciprocal lattice mapping. *Appl. Phys. Lett.* **100,** 071901 (2012).
8. Bouvier, P. & Kreisel, J. Pressure-induced phase transition in LaAlO3. *J. Phys. Condens. Matter* **14,** 3981 (2002).
9. Biegalski, M. D. *et al.* Critical thickness of high structural quality SrTiO3 films grown on orthorhombic (101) DyScO3. *J. Appl. Phys.* **104,** 114109 (2008).
